# In Vivo Genome Editing as a Therapeutic Approach

**DOI:** 10.3390/ijms19092721

**Published:** 2018-09-12

**Authors:** Beatrice Xuan Ho, Sharon Jia Hui Loh, Woon Khiong Chan, Boon Seng Soh

**Affiliations:** 1 Disease Modeling and Therapeutics Laboratory, A*STAR Institute of Molecular and Cell Biology, 61 Biopolis Drive Proteos, Singapore 138673, Singapore; xbho@imcb.a-star.edu.sg (B.X.H.); lohjh@imcb.a-star.edu.sg (S.J.H.L.); 2 Department of Biological Sciences, National University of Singapore, Singapore 117543, Singapore; dbscwk@nus.edu.sg

**Keywords:** in vivo, genome editing, correcting genetic mutations, ZFN, TALENs, Cas9

## Abstract

Genome editing has been well established as a genome engineering tool that enables researchers to establish causal linkages between genetic mutation and biological phenotypes, providing further understanding of the genetic manifestation of many debilitating diseases. More recently, the paradigm of genome editing technologies has evolved to include the correction of mutations that cause diseases via the use of nucleases such as zinc-finger nucleases (ZFN), transcription activator-like effector nucleases (TALENs), and more recently, Cas9 nuclease. With the aim of reversing disease phenotypes, which arise from somatic gene mutations, current research focuses on the clinical translatability of correcting human genetic diseases in vivo, to provide long-term therapeutic benefits and potentially circumvent the limitations of in vivo cell replacement therapy. In this review, in addition to providing an overview of the various genome editing techniques available, we have also summarized several in vivo *genome* engineering strategies that have successfully demonstrated disease correction via in vivo genome editing. The various benefits and challenges faced in applying in vivo genome editing in humans will also be discussed.

## 1. Introduction

Genome editing, commonly known as genetic engineering, is widely used in biological research. Genome editing strategies involve DNA modification, through insertion, deletion, or replacement of defective DNA in live organisms and have been widely established over the last few decades. Genome editing exploits endogenous DNA repair machinery through the use of common engineered nuclease-based platforms that introduce a targeted double stranded break (DSB). This includes meganucleases, zinc-finger nucleases (ZFN), transcription activator-like effector nucleases (TALENs) and more recently, clustered regularly interspaced short palindrome repeats (CRISPR) have been successfully adapted through the use of RNA-guided endonucleases known as Cas9, which can easily target virtually any genomic location [1,2,3]. At present, two main approaches for gene editing therapies have been characterized: (i) in vivo genome editing, which may consist of either viral or a combination of viral vector and lipid nanoparticles to deliver therapeutic components, that may also constitute protein or mRNA-based delivery of a genome editing cargo [4] or (ii) ex vivo gene therapy, which consists of transduction of the therapeutic gene into patient-derived somatic cells, followed by subsequent transplantation back into the recipient.

Genome engineering techniques have often been associated with disease modeling—either to generate mutations or the isogenic control cell line using patient derived iPS cells. With the advancement in gene editing technologies in recent years, in vivo corrections of defective gene holds great promise as a therapeutic approach to treat human genetic diseases.

In this review, we briefly summarize the different techniques widely used in genome editing, and some of the vector delivery systems that act as a vehicle for in vivo delivery. We also highlight some of the studies where in vivo genome editing has been successfully employed to treat genetic diseases, and discuss its utility, impact, and limitations on translational medicine going forward.

## 2. Gene Editing Technologies

### 2.1. Homologous Recombination (HR)

**Figure 1:**
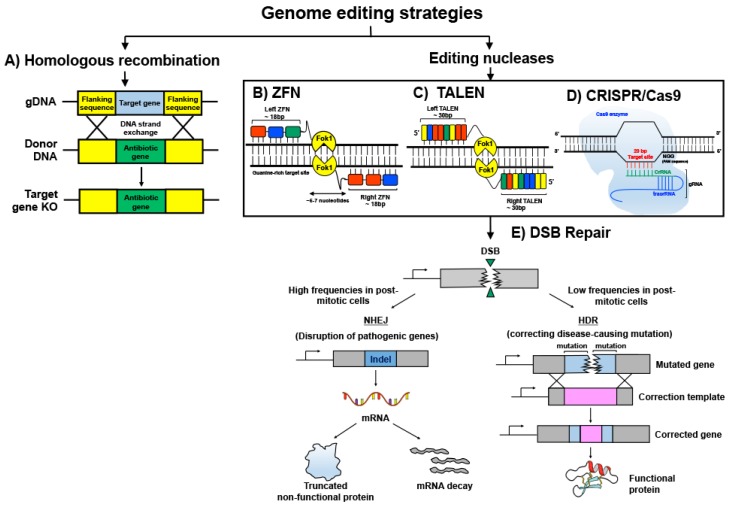
Genome editing strategies. Genome editing technologies can be employed using non-enzymatic and enzymatic methods. (**A**) Homologous recombination is a process whereby DNA strand exchange occurs between two homologous DNA molecules. The antibiotic gene in the donor DNA serves as a selection marker to ascertain the knockout of the target gene in the cell. (**B**) Zinc finger modules are linked together in tandem using conserved linker sequences to recognize target sequences that are composed of DNA triplets. Fok1 nuclease is fused to the zinc finger array. (**C**) transcription activator-like effector nucleases (TALEN) are similar to zinc-finger nucleuses ZFN but confer more flexibility by having each transcription activator-like effector (TALE) module recognizing only one nucleotide base. TALE modules can be joined together to from tandem arrays that bind the target site. (**D**) CRISPR/Cas9 system utilizes a custom-made gRNA (guide RNA) to transport Cas9 nuclease to the target gene. (**E**) ZFN, TALEN, and CRISPR/Cas9 methods result in a nuclease-induced DNA double-stranded break at the target site that stimulates the DNA repair mechanism: non-homologous end joining (NHEJ) or homologous directed repair (HDR). NHEJ induces error-prone repair by bluntly joining the cleaved DNA strands together. Hence, it is useful in disrupting pathogenic mutations through indel disruption of the target gene. HDR confers high-fidelity correction of the pathogenic mutation by using a repair DNA template to drive homologous recombination. It is important to note that NHEJ is active in both proliferative and non-proliferative cells, but HDR is restricted to proliferative cells only. (**B**): A ZFN triplet module is depicted using colored blocks. The varying colors differentiate ZFN triplets of different base combinations. (**C**): Different DNA bases are depicted as Yellow, Blue, Red and Green colored blocks.

The exchange of nucleotide sequences between two similar DNA molecules is termed as homologous recombination (Figure 1A). It is the earliest genome editing technique that utilizes an innate cellular process to artificially induce site-specific mutations. Essentially, foreign DNA molecules containing homologous arms to that of the flanking regions of the target gene in the host genome are the pre-requisite for stimulating homologous recombination. While the length of the homologous arms at each side of the DNA fragment varies among species and directly affects the frequency of recombination events, mammalian cells generally require more extensive stretches of homology (~1.2 kbp) than bacteria (~20 bp) for efficient recombination, due to their relatively larger genomic size [1,2,3].

Rad51 protein plays an important role in initiating homologous recombination in mammalian cells by binding to a single strand of the foreign DNA at the 3′ end and allowing it to invade the host genome to scan for sequence homology. Once extensive homologous base-pairing occurs, the complementary strand of the host DNA is displaced to create a single strand cross-over known as the Holliday junction [4]. Endonuclease GEN1 cleaves the Holliday junction and the foreign DNA and host genome are fused together by ligase [5]. “Donor DNA template containing the gene of interest could be fused with a drug resistance gene or fluorescent marker to enable the selection of transformants that possess the desired gene modifications (Figure 1A)”.

### 2.2. Zinc-Finger Nucleases (ZFN)

Zinc finger proteins have specific DNA binding properties and function naturally as transcription factors in eukaryotes to regulate expression of target genes [6,7]. They consist of approximately 30 amino acids in a ββα conformation that is folded into a finger-like structure through the interactions of the Zn^2+^ atom and two cysteine and two histidine residues [8,9,10]. Amino acid within the zinc finger can be modified to interact with specific DNA bases in multiples of three [11,12]. Engineering a tandem array of zinc finger modules using conserved linkers that recognizes an 18 bp sequence can thus allow various DNA sequences to be targeted and a conferment of specificity within 4^18^ bp (Figure 1B). Zinc finger arrays can be additionally fused with nucleases, such as endonuclease Fok1, to induce alterations of the genome at targeted sites [13,14]. The non-specific catalytic domain of nucleases is commonly used to increase the variety of DNA sequences to be altered. Since FokI requires dimerization of its subunits to initiate DNA cleavage, two distinct zinc finger arrays with each coupled to a FokI nuclease domain are designed to bind opposite DNA target strands with an intervening spacer region of 5–7 nucleotides [10,15]. Dimerization of FokI domains attached to each zinc finger nuclease module leads to a DSB of the target DNA, leading to a series of potential cellular repair mechanisms that could be leveraged to induce genome sequence revisions [10,16]. Although zinc fingers are theoretically convenient and useful, stitching zinc fingers together is time-consuming and limited to sequences composed of triplets.

### 2.3. Transcription Activator-Like Effector Nucleases (TALEN)

Transcription activator-like effector (TALE) protein is derived from bacteria plant pathogen *Xanthomona.* It has a DNA binding module consisting of tandem repeats of about 34 amino acids per repeat. Each repeat contains two variable di-residues (RVD) at amino acid position 12 and 13 that confer DNA base recognition [17]. Similar to zinc finger nucleases, the DNA binding domains of TALE can be synthesized to form an array that recognizes a sequence specific target and engineered with a nuclease domain, usually FokI nuclease, for target gene modifications [18,19] (Figure 1C). Each TALE DNA binding module recognizes one base pair and thus offers more flexibility in the selection of target regions compared to triplet-confined zinc fingers. The functionality of TALEN is similar to ZFN whereby FokI subunits dimerize to introduce DSB of the target gene. However, an intervening spacing of 10–30 bp is required between the two bound TALEN arrays for DNA cleavage to occur efficiently. Previously, the selection of TALE was limited to target sequences containing 5′-T. However, this has been overcome by using mutant variants at TALE N-terminal domains that can recognize and bind to any DNA bases [20,21] A significant advantage of using TALEN over zinc finger nucleases is the abrogation of engineering linkages as binding efficiencies of DNA domains have known to be influenced by these intervening additional linkage sequences. Previously, cloning of TALEN arrays possess great technical challenges due to the many repeat sequences present in each TALE monomer. However, in recent years, techniques such as the ‘Golden Gate’ cloning system have been developed to enable rapid and ease of assembling TALEN arrays [22].

### 2.4. CRISPR/Cas

CRISPR/Cas is a prokaryotic adaptive immune system that incorporates foreign pathogenic DNA, such as from bacteriophages, into the bacteria genome to prevent future infections. The mechanism involves a CRISPR gene array consisting of foreign DNA elements integrated between short palindromic repeats being transcribed into RNA fragments (crRNA) that correspond to the foreign DNA. A bacterial scaffold RNA known as the trans-activating crRNA (tracrRNA) is also being transcribed to assist in the association of crRNA with Cas9, which in turn guides the endonuclease to its complimentary DNA target site [23] (Figure 1D). The discovery of this system revolutionized genome editing strategies whereby a guide RNA (gRNA) comprising of a customized crRNA sequence and tracrRNA fused together can be used to re-direct Cas9 towards any target of interest [24]. However, a protospacer adjacent motif (PAM) of sequence 5′-NGG-3′ must succeed the target DNA for the recognition and binding of Cas9. An exceptionally beneficial feature of CRISPR/Cas system compared to other genome modification strategies is the ability to multiplex, allowing several genomic sites to be revised simultaneously [25] This powerful technique opens up huge future possibilities of correcting mutations in polygenic diseases such as cardiovascular disorders, to reverse illnesses.

## 3. In Vivo Applications of Genome Editing Tools in Pre-Clinical Models

Whilst earlier studies have demonstrated the hope of cell replacement as a therapeutic option for diseases such as retinal dystrophy, the limitations faced with consistent need for cell transplant therapy could be eliminated through in vivo genome editing. Table 1 summarizes the more recent advancements of using in vivo genome editing as a therapeutic tool in pre-clinical animal models.

### 3.1. ZFN for In Vivo Genome Editing Therapeutics

#### 3.1.1. Cancer Diagnosis and Therapy

Gene therapy in cancer presents a promising approach to modify, delete, or replace the genes of target cells causing tumorigenecity, these cell types include circulating tumor cells, dormant stem cells, and even T-lymphocytes or dendritic cells [62]. Due to the prevalence of drug resistance, gene therapy may be designed in accordance to an individual’s needs, based on an individual’s genetic constituents, as well as tumor specifics, genetic and host immune response, hence, aimed at providing optimal efficacy. While the HR repair pathway is functionally important for repairing DNA double stranded breaks, cancer cells exploit this pathway to avoid apoptosis. More recently, Chen et al. have demonstrated that delivery of the lentiviral vector containing promoter XRCC2 to target cancer-specific tumor cells with hyperactive HR genes holds great promise for in vivo tumor prognosis and therapy [63]. Lentivirus bearing pXRCC2-luciferase vector was shown to enhance in vivo imaging of the bioluminescence signal, which proved to be significantly greater in xenograft implanted mice, as compared to control cancer-free mice. In addition, XRCC2 promoter driving firefly luciferase or diphtheria A (DTA) gene injected subcutaneously to HeLa xenograft mice model demonstrated that pXRCC2-DTA lentivirus significantly inhibited the growth of HeLa xenografts. Hence, this reemphasized the potential of viral-mediated delivery pXRCC2-DTA constructs as an effective tool for attenuating tumor growth in vivo [63].

Well established proof-of-principle ZFN-mediated in vivo animal models have made their way towards clinical trials for the treatment of hemophilia B, HIV, and Mucopolysaccharidosis (MPS) [33,64]. These studies are further elaborated in this section.

#### 3.1.2. Hemophilia

Hemophilia is a genetic disease of the liver that is caused by deficiency of blood coagulation genes, including *factor* VIII, and *factor* IX [29]. The genetic defect impairs the body’s ability to form blood clots, resulting in prolonged duration for coagulation, although current clinical interventions have incorporated infusion of clotting factors as remedies for the symptoms of hemophilia, these strategies do not provide a permanent cure. Going beyond intravenous infusion of recombinant FVIII and FIV, ZFN-mediated in vivo editing utilizing AAV vector delivery system may be performed in a tissue specific manner vector delivery system may be performed in a tissue specific manner [26,27].

The authors highlighted the clinical relevance of ZFN-mediated gene correction, as presented by improved clotting times in a humanized mouse model of hemophilia B injected with ZFN adeno-associated virus donor template vector [26]. Co-delivery of ZFN and a donor template in vivo resulted in clinical improvements as observed by significantly increased circulating human coagulation factor IX (FIX) [26]. ZFN-mediated in vivo genome editing presents a plausible approach for permanent genome correction of the defective *F9* gene. Genetic manipulation produced an effect sufficient to reverse phenotypic defect previously observed in Hemophilia B mice, hence demonstrating the therapeutic potential of AAV/ZFN-mediated genome editing to treat liver disease in non-replicating cells [28].

#### 3.1.3. Mucopolysaccharidosis II (MPS II) or Hunter’s Syndrome

Mucopolysaccharidosis II (MPS II) is a rare metabolic disease in which the affected gene is on the X chromosome. The X-linked recessive lysosomal disorder results from iduronate 2-sulfatase (IDS) deficiency, leading to the accumulation of glycosaminoglycans (GAGs) in various body tissues and causing disease. Current therapeutic approaches involve bone marrow transplantation and enzyme replacement therapy (ERT) [65]. The latter requires long-term intravenous (IV) infusion enzyme infusion with idursulfase (Elaprase) to manage the disease, but does not eliminate disease progression, hence leading to current research advancements that motivated to circumvent the limitations of ERT and prevent the onset of neurological disease arising from MPS II [66].

Over several years, adeno-associated virus (AAV) vectors have shown increasing clinical promise as a therapeutic tool for gene therapy in treating patients with haemophilia B and congenital blindness [67,68] This motivated the approach of ZFN-mediated in vivo genome editing of the albumin locus in hepatocytes as a platform for protein replacement therapy [27,32] While ZFN-mediated site-specific insertion to target the disease locus itself may not be significantly effective in treating the disease phenotype, Laoharawee and colleagues demonstrated that the targeted insertion of a therapeutic transgene into a highly transcriptionally active locus ensures the therapeutic gene will be effectively replicated during cell proliferation, therefore allowing for sustained long term expression of the protein of interest [32]. In addition, pre-clinical studies showed high-levels of enzyme expression of hIDS activity in the liver was capable of achieving 200-fold higher levels as compared to wild-type mice. A proof-of-concept study also revealed hIDS activity in the liver promoted secretion and systemic distribution into the bloodstream [32].

Similarly, in a different study, Sharma and colleagues presented an in vivo genome editing approach as a platform for protein replacement therapy [27]. The authors reported a technique of ZFN-mediated site-specific integration of therapeutic transgene within the albumin gene in the liver. A pre-clinical mouse model of hemophilia A and B receiving AAV vector delivery in vivo displayed long term expression of human factors XIII and IX, that achieved therapeutic levels [27]. More recently, Sangamo Therapeutics demonstrated the use of in vivo genome editing in an open-labeled clinical trial that evaluates single ascending IV doses of Sangamo’s SB-913, a zinc finger nuclease (ZFN)-mediated gene editing therapy for MPS II or Hunter’s syndrome [69].

### 3.2. TALENS for In Vivo Genome Editing Therapeutics

#### Papillomavirus-Related Malignant Neoplasm

The majority of high-risk human papillomavirus (HPV) are causative agents in tumors of the anogenital and oropharyngeal regions, as well as other regions such as head and neck cancers. Tumorigenesis occurs as a result of HPV DNA genome integration into the host cell genome, therefore, it favors cancer cell growth and viability. Pathogenesis of papillomavirus-related malignancies has been associated primarily from deregulated expression of two viral oncoproteins, termed E6 and E7 [70,71,72]. It has been suggested that E6 induced degradation of the cellular tumor suppressor P53, whereas E7 causes retinoblastoma (Rb) protein destabilization. Hence, motivated the use of bacterial CRISPR/Cas RNA-guided endonuclease to target and cleave the E6 and E7 gene in cervical carcinoma cells induced by HPV, resulting in cell cycle arrest and eventual cancer cell death [72]. As reviewed by Lau and Suh, the safety and efficacy of TALEN-mediated plasmids for treatment of HPV-related cervical intraepithelial neoplasia has been undergoing Phase I trials in an open-label three-cohort study [73]. The research aims to disrupt HPV E6/E7 DNA, leading to the induction of p53 or Rb, respectively [72,73].

### 3.3. CRISPR-Cas9 for In Vivo Genome Editing Therapeutics

#### 3.3.1. Human Immunodeficiency Virus (HIV-1)

The fatal outcome of viral replication as well as absence of effective treatment presents the need for the discovery of novel therapeutic approaches to cure this infection. Although current anti-retroviral therapy (ART) remains effective against reduction of viral load in patients and delays disease progression, the latent viral reservoir remains insensitive to ART and not detected in the immune system. Due to the complexity of HIV, HIV-1 infected individuals require life-long therapy using ART, in addition, these drugs are insensitive towards the latent viral reservoir that is established shortly after an infection [74]. The potential for endonuclease-based gene editing in vitro demonstrated by various groups have led to current investigations of endonuclease-specific targeting as a means of in vivo therapy [75].

For the first time, HIV-1 provirus replication was demonstrated to be eliminated from infected cells in three different HIV-1 transgenic humanized mice models carrying the provirus [41]. Through the use of AAV vector to deliver single-guide RNA (sgRNAs) plus Staphylococcus aureus Cas9 (saCas9) (sgRNA/saCas9) via intravenous (IV) injection, intravenously injected sgRNAs/saCas9 AAV displayed: (i) excised proviral DNA and significantly reduced RNA expression in several tissues of Tg26 mice, (ii) reduced systemic EcoHIV infection in EcoHIV acutely infected mice and (iii) efficient viral excision in humanized bone marrow/liver/thymus (BLT) mice with chronic HIV-1 infection [41]. This research displayed a proof-of-principle where in vivo excision of HIV-1 proviral DNA in latently infected human cells has the potential to be used as a tool to delete the targeted fragments of the HIV-1 genome, therefore, preventing and improving prognosis in HIV1 diseases. Similarly, AAV vector expressing gRNA/saCas9 demonstrated in vivo eradication of HIV-1 DNA in various cells and tissues upon CRISPR/Cas9 delivery, resulting in significant decrease in circulating blood lymphocytes [42]. 

#### 3.3.2. Duchenne Muscular Dystrophy (DMD)

Duchenne muscular dystrophy is caused by the mutation of the dystrophin-encoding gene that is responsible for maintaining muscle integrity. The clinical phenotype of DMD in patients carrying this genetic mutation results in progressive muscle degeneration, causing muscle weakness and myopathy. Over several decades, different approaches from gene- to cell-based therapies have been developed in attempts to treat patients with DMD. These treatments typically focus on the delivery of functional *Dmd* alleles or dystrophin-like proteins to patients. However, therapeutic challenges faced with an absence of clinically effective treatments and the advancement of genome editing in postnatal somatic cells have motivated researchers to investigate a possible approach to alter genomic DNA in vivo. Much success has been achieved by three independent research groups, demonstrating that CRISPR/Cas9-mediated genome editing was able to correct the defective dystrophin gene in in vivo mice models of DMD, which was shown by improved muscle structure and function [48,49,50] For instance, in order to demonstrate proof-of-concept that correction of the disease causing mutation for DMD can restore dystrophin expression in vivo, Long et al. utilized a postnatal *mdx* mice model harboring a frame shift mutation in exon 23 of the gene. The rescue of developing phenotype associated with DMD after CRISPR/Cas9-mediated gene correction reflects a clinical advantage of correcting the primary genetic lesion responsible for DMD. This was further demonstrated through fusion of genetically corrected satellite cells with dystrophin fibers, allowing for the regeneration of dystrophin muscle [49].

#### 3.3.3. Amyotrophic Lateral Sclerosis (ALS)

Amyotrophic lateral sclerosis caused by loss of motor function is a fatal and incurable neurodegenerative disease that affects the spinal cord and brain. Approximately 20% of familial cases of ALS arise from autosomal dominant mutations in the superoxide dismutase 1 (SOD1) gene [52]. The study conducted by Gaj et al. demonstrated disruption of mutant SOD1 expression in the G93A-SOD1 mouse model of ALS through in vivo CRISPR-Cas9 genome editing using an AAV vector can result in delayed disease onset, improved motor function, and reduced muscle atrophy [52]. The authors concluded that CRISPR-mediated genome editing can potentially be used as a therapeutic approach for familial ALS as well as disorders associated with the central nervous system (CNS) that are caused by autosomal dominant mutations. Hence, genome editing tools that permanently correct the genetic mutation found in patients may present a promising approach for treating neurodegenerative disease. Whilst studies have also highlighted the therapeutic benefits of AAV delivered artificial microRNA showed extended survival and delays paralysis in the mice model of ALS, microRNA presents a therapeutic approach that requires long-term therapy [76].

#### 3.3.4. Hereditary Tyrosinemia Type I (HTI)

Another well-demonstrated proof-of-concept study was reported by Yin and colleagues who have highlighted the potential for CRISPR-Cas9 mediated genome editing in a mice model of HTI [36]. HTI is a fatal genetic disease caused by a mutation in the last enzyme of the catabolic pathway, fumarylacetoacetate hydrolase (FAH). FAH deficiency is associated with accumulation of fumarylacetoacetate where build up of toxic metabolites in hepatocytes results in severe liver damage, as well as renal proximal tubule damage. *Fah5981SB* mouse model harboring a homologous G to A point mutation of the last nucleotide on exon 8 fully recapitulates the human disease [77]. Point mutation in this region of the human genome causes exon 8 skipping during splicing, resulting in the production of a truncated and unstable protein.

A recent study by Yin et al. has shown that the application of a combination of viral and lipid nanoparticle-mediated delivery of mRNA Cas9, guide RNA, and repair template DNA was capable of inducing specific genomic correction to the Fah-splicing mutation in an in vivo *Fah5981SB* mouse model. The treatment resulted in the highly efficient (>6%) generation of (Fah)-positive hepatocytes with functional correction of disease symptoms such as weight loss and liver damage [37]. The stable genetic repair in *Fah* deficient cells further resulted in its’ expansion and repopulation in the liver.

## 4. Summary of Some Current Vector Delivery Systems

Vector-mediated delivery of therapeutic agents remains a promising tool to achieve in vivo genome editing to cure both human genetic and acquired diseases. Several studies have demonstrated that the use of viral-mediated vector systems to deliver target genes to specific affected tissues or cells can attain highly efficient transfection frequencies with minimal cytotoxicity. On the other hand, many have also been investigating the possibility of utilizing non-viral vectors to overcome the major drawback related to safety faced with the use of viral vectors. This section will be summarizing some of the current viral vector delivery systems used in vivo, as well as current proteins and nanoparticles that have been established as efficient vectors for in vivo and in vitro transformations.

### 4.1. Adeno-Associated Viral (AAV) Vector

The recombinant AAV (rAAV)-mediated vector system is an established technique for efficient genome transduction and double-strand break (DSB) repairs that infect both dividing and non-dividing human cells [78]. Earlier studies demonstrated that recombinant proteins encoded in AAV vectors facilitate homologous recombination between viral and chromosomal sequences in rodent and human cells; hence promoting its transduction efficiency [79,80,81] Furthermore, despite the low presence of multiplicity of infection (MOI), rAAVs has been shown to display a high frequency of gene targeting, suggesting that the combination of rAAV vectors with DSBs is a promising strategy to stimulate genomic targeting [82].

### 4.2. Lentiviral Vector

Lentivirus, a subset of retroviruses, is commonly known for its efficient transduction and integration into host cells. This lentiviral-mediated vector creates an effective system for the delivery of a transgene into non-dividing cells and acts as an efficient shuttle for large genetic material that maintains stable long-term transgene expression. The use of lentiviral vectors for long-term therapeutic benefit has been indicated through earlier studies of effective treatment in pre-clinical animal models with neurological disorders such as Parkinson’s disease, Huntington’s disease, Alzheimer’s disease and spinal cord injuries, thus suggesting the potential of this vector for gene therapy in patients [83,84,85,86,87].

### 4.3. Supercharged Proteins

The limitation faced with proteins as a therapeutic tool lies with its’ inability to penetrate mammalian cells. Supercharged proteins are proteins possessing highly positive or negative net charges that are capable of penetrating mammalian cells both in vitro and in vivo. Recently, Zuris et al. showed highly efficient Cas9-mediated genome editing using cationic lipid vectors in the inner ear hair cells of an in vivo mice model [88]. Their results suggest the potential for cationic lipids to act as a delivery system for proteins in in vitro and in vivo studies. While this technology appears promising, specific cell targeting and nuclear localization mechanisms need to be further evaluated before such an approach can be clinically relevant.

### 4.4. Nanoparticles (NPs)

Lipid nanoparticles may be used for the efficient delivery of nucleases, guide-RNA, and template DNA in a single vector for CRISPR/Cas9. Zhang et al., constructed a novel polyethylene glycol phospholipid-modified cationic lipid nanoparticle (PLNP)-based delivery system that facilitated the delivery of an intratumor injection of Cas9/sgPLK-1 plasmids into tumor bearing-mice that resulted in an observed suppression of tumor growth in vivo [89]. Despite the relatively less efficient genomic transduction observed by NPs, Han and colleagues have observed comparable gene expression levels of NPs to AAV vector delivery systems [90]. In addition, NPs with the ability to transduce dividing and non-dividing cells have also been associated with the absence of cytotoxicity in retinal pigment epithelium (RPE) cells [91].

## 5. Advantages and Limitations of In Vivo Gene Editing

In vivo gene editing techniques involve the introduction of either a DNA-based nuclease expression system or direct delivery of the programmable nuclease. The high expression of editing nuclease in a DNA-based editing system increases the risks of nuclease induced off-target mutagenesis compared to protein delivery systems where the dosage levels of nuclease can be controlled. Viral vectors, such as adeno-associated viruses (AAV), are popularly used to deliver the editing system in vivo [92]. They are advantageous because they come in a variety of serotypes that permit high delivery efficacy for various tissue types and can infect proliferative and non-proliferative cells [93,94]. The non-integrative property of viral DNA into the host genome of AAV is an added safety feature compared to integrating lentiviral vehicles. The main drawback of using AAV is their limited cargo capacity that could hold a maximum of 4.7 kb. ZFNs possessing small protein size and the dimeric pair could be easily packaged into AAV [95]. However, a single AAV would not be able to contain gRNA and Cas9, given that the latter already occupies 4.2 kb. To incorporate CRISPR/Cas9 system, two AAV vectors are often required to ferry gRNA and Cas9 separately [10].

Editing rates for the current nuclease platforms (ZFN, TALEN and CRISPR/Cas9) are dependent on the type of delivery methods used, target cell type, fitness of target cell after genetic manipulation and the number of target cell to nuclease ratio. Increasing viral loads to deliver higher amounts of editing nucleases may be a direct way to enhance editing rates but it also imposes substantial risks of deleterious off-target gene cleavage, impairing functions, and inducing oncogenic potential to target cells. Furthermore, it is also possible that immunotoxicity might arise when high concentrations of microbial-derived peptides of programmable nucleases (enzymatic and DNA binding domains) are presented by Major Histocompatibility Complex (MHC) Class I molecules [10,96].

To date, all in vivo gene editing techniques face the common challenge of controlling the distribution of editing nuclease. Though AAV has been reported to be useful vectors, they are still restricted to organ systems where transduction is clinically efficient such as the eye, brain, liver, and muscles [93,97,98]. To circumvent this limitation, non-viral delivery systems are currently under development to expand the range of targetable tissues and reduce potential safety risks [8].

Although in vivo therapies have considerable limitations, it does confer some advantages over in vitro therapies. Directly revising the genome in native tissues in vivo can be beneficial especially for cell types, such as neurons, that are either less likely to survive after genetic amendments or lose their function when artificially grown in in vitro environment. It also obviates the problem of possible poor engraftment of edited cells, which is frequently encountered in in vitro editing therapies. Additionally, in vivo gene editing strategies have the potential to induce genetic corrections in multiple tissue types depending on the delivery mode chosen. This potentially allows in vivo gene editing strategies to be used in the treatment of a range of diseases and multi-systemic diseases, such as HIV [42].

Rewriting the genome in vivo has made significant progress over the years. Gene editing strategies utilizing AAV serotype encoding ZFN has shown the most promising results amongst other editing nuclease platforms. It was demonstrated to be successful in treating hepatic dysfunctions in hemophilia B and hereditary tyrosinemia mouse models and phase I clinical trials are underway for the treatment of human HIV and Hunter’s syndrome [26,36,99]. Current gene manipulation techniques differ from one another in several ways (target site recognition sequence, protein size, and difficulty of engineering) and have their respective pros and cons (summarized in Table 2). More work is required to improve the safety of delivery systems, gain better control in viral dosage units and distribution, and increase gene editing efficiencies in desired cell types in order for in vivo gene editing methodologies to be employed in clinical medicine.

## 6. Conclusions

With the advancement in genome editing technologies, correcting genetic mutations in targeted tissues and cells to cure the genetic cause of a disease has recently reemerged as a therapeutic modality that holds great promise. As summarized in this review, various studies have shown that the correction of a genetic mutation in the DNA results in delayed disease progression or presents a protective effect against disease manifestation—illustrating a proof-of-concept for in vivo genome editing.

Several groups have shown that the possibility of mediating in vivo genome editing through programmable nucleases (as mentioned in Section 2) may have therapeutic potential with several successes demonstrated by CRISPR/Cas9 AAV vector-mediated in vivo delivery resulting in improved clinical phenotype, as observed in several studies [49,50,54,100]. However, these strategies for precise engineering and delivery of gene-editing mediated endonucleases are still faced with many obstacles that have yet to be overcome before genome editing may be taken into the clinic. For example, clinical applications of in vivo AAV vector delivery are often faced with limitations such as large sizes of constructs, negative charge, and low membrane permeability [101]. Moreover, although CRISPR-Cas9 genome editing presents an efficient and reliable technique for targeted genome engineering in living cells, the implications of human genetic variation may potentially result in a substantial inter-individual response to Cas9 endonucleases for in vivo genome therapy. This suggests the need to provide prescreening procedures such as whole-genome sequencing for patients prior to receiving in vivo genome therapy [102]. Prescreening would enable clinicians to understand the genetic profile of patients and therefore allow the design of specific CRISPR-based therapies to provide optimal efficacy, an accurate safety profile, and prevention of drug-related adverse events. More recently, significant on-target mutagenesis, large deletions, and DNA breaks introduced through gRNA/Cas9 have been reported to result in deletions extending over many kilobases [103]. Furthermore, several studies revealed complexities in using CRISPR/Cas9 genome editing that resulted in deletions, inversions, and duplications in the DNA [104,105].

A major challenge of the use of in vivo genome editing is the delivery of these therapeutic agents into the central nervous system (CNS). The presence of the blood brain barrier (BBB) hinders the delivery of therapeutics to various cell types in the CNS, in particular to the brain and spinal cord for treatment of neurodegenerative diseases such as amyotrophic lateral sclerosis (ALS) or spinal muscular atrophy (SMA). Due to the highly hydrophobic nature of the BBB, it restricts the penetration of delivery systems depending on their hydrophobicity. Hence, therapeutic delivery agents have to be highly lipid and soluble to ensure the active diffusion of these compounds across the membrane of the BBB. Interestingly, early studies have shown that the intravenous administration of AAV9 resulted in the preferential targeting of neurons and astrocytes in neonatal and adult mice, respectively [106]. Furthermore, it was demonstrated by two independent groups that the IV injection of AAV9 was capable of crossing the BBB and was transduced across the motor neurons within the spinal cord and the sensory fibers and surrounding cells in the CNS [106,107].

The advancements of non-invasive therapeutic strategies for treating neurodegenerative diseases raises the hope for mutant gene correction in patients with spinal cord motor neuron disorders and overcomes the requirement for direct intracerebral injections that involve invasive surgical procedures. In a study conducted by Gaj et al., G93A-SOD1 treated mice displayed delayed disease onset, prolonged survival, improved rotarod performance, and maintained or gained weight [52]. However, AAV-mediated delivery of therapeutic reagents has as its shortfall no delay in disease progression (upon onset of disease), which had been observed between the treated and untreated groups [52]. The authors hypothesized that this may be due to insufficient genome editing in the astrocytes, which was shown by the low Cas9 expression in the gray and white matter astrocytes; hence suggesting that the level of AAV vector transduction could be limited.

All in all, clinical trials and in vivo animal studies are currently ongoing to investigate and pursue the clinical applicability and translatability of in vivo genome editing in order to achieve a permanent cure for monogenetic diseases. With the current advances in in vivo genome editing, the advantages of using it as a platform for therapy significantly outweigh these limitations. It is hopeful that these challenges will be overcome to steer us towards a next-generation therapeutic approach to treat a wide range of diseases including those that currently do not have a cure, such as HIV.

## Figures and Tables

**Table 1:** Summary of recent advancements of in vivo pre-clinical models as a therapeutic approach.

Strategies	Disease	Model	Reference
ZFN	Hemophilia A and B	Mouse	Li et al., 2011 [26] Sharma et al., 2015 [27] Anguela et al., 2013 [28] Park et al., 2016 [29]
	Human immunodeficiency Virus (HIV-1)	Human	Holt et al., 2010 [30]
	Mucopolysaccharidosis II (MPS II) or Hunter’s syndrome	Mouse	Laoharawee et al., 2016 [31] Laoharawee et al., 2018 [32] Sawamoto et al., 2018 [33]
Transcription activator-like effector nucleases (TALENS)	Human immunodeficiency Virus (HIV-1)	Humanized Mouse	Benjamin et al., 2016 [34]
	Hepatitis B Virus (HBV)	Mouse	Weber et al., 2013 [35]
CRISPR-Cas9	Hereditary Tyrosinemia Type I (HTI)	Mouse	Yin et al., 2014 [36] Yin et al., 2016 [37] Pankowicz et al., 2016 [38]
	Human immunodeficiency Virus (HIV-1)	Human	Ebina et al., 2013 [27] Dai et al., 2016 [39]
	Human immunodeficiency Virus (HIV-1)	Primary CD4+ T cells	Liu et al., 2017 [40]
	Human immunodeficiency Virus (HIV-1)	Humanized Mouse	Yin et al., 2017 [41] Kaminski et al., 2016 [42]
	Age-related Macular Degeneration (AMD)	Mouse	Kim et al., 2017 [43] Kim et al., 2017 [44]
	Retinal Dystrophy	Rat; Ex vivo	Yanki et al., 2017 [10]
	Retinitis Pigmentosa	Mouse	Latella et al., 2016 [45] Wu et al., 2016 [46] Zhu et al., 2017 [47]
	Duchenne Muscular Dystrophy (DMD)	Mouse	Tabebordbar et al., 2016 [48] Long et al., 2016 [49] Nelson et al., 2016 [50] Bengtsson et al., 2017 [51]
	Amyotrophic Lateral Sclerosis (ALS)	Mouse	Gaj et al., 2017 [52]
	Haemophilia B	Mouse	Singh et al., 2018 [53]
	Rett syndrome	Mouse	Swiech et al., 2015 [54]
	Ornithine Transcarbamylase (OTC) deficiency	Mice	Yang et al., 2016 [55]
	Alpha-1 antitrypsin (AAT) deficiency	Mouse	Song et al., 2018 [56]
	Cancer	PD-L1+ Tumor xenograft model (in vivo); Human	Rupp et al., 2017 [57] Castillo et al., 2016 [58]
	Leber’s congenital amaurosis (LCA)	Human	Bainbridge et al., 2008 [59] Maguire et al., 2008 [60] Ruan et al., 2017 [61]

**Table 2:** Applications of the technology in ZFN, TALEN, and CRISPR/Cas9 gene editing.

	ZFN	TALEN	CRISPR/Cas9
Recognition/target site	18–36 bp/zinc finger pair; guanine-rich region	30–40 bp per TALEN pair	22 bp; followed immediately by 5′-NGG-3′ PAM sequence
Targeting specificity	18 bp ZFN can confer specificity within 4^18^ bases [10]	TALEN plasmid library developed can target 18,742 human genes	Unknown; theoretically any genomic site that precedes PAM sequence
Off-target mutagenesis	Unknown and hard to determine mutagenic sites due to many possible indiscriminate protein-DNA interactions that can occur	Unknown and hard to determine mutagenic sites due to many possible indiscriminate protein-DNA interactions that can occur	Easier to predict possible mutagenic sites by utilizing Watson–Crick base-pairing rules
Ease of Delivery	Difficult due to extensive cloning needed to link zinc finger modules together	Difficult due to extensive TALE repeat sequences	Easy, facile design of gRNA and standard cloning techniques
Methods employed to deliver editing systems in vivo	AAV	AAV	AAV Lentivirus
Multiplexing ability	No	No	Yes
Clinical or pre-clinical stage	Clinical trial application for HIV and Hunter’s syndrome	Pre-clinical	Pre-clinical
Advantages	Small protein size (<1 kb) allows packaging into a single AAV	High specificity with each module recognizing 1 bp; no need to engineer linkage between repeats	Enables multiplexing (targeting multiple genes)
Limitations	Length of target sequence confined to the multiples of three; cumbersome cloning methods that needs additional linker sequences to fuse modules together	Large protein size makes it challenging to utilize viral system; repetitive sequences may induce undesirable recombination events within the TALE array	Limited PAM sequences in human genome; Cas9 nuclease (~4.2 kb) is large for packaging into AAV

## Abbreviations

ZFN	Zinc-finger Nucleases
TALEN	Transcription Activator-like Effector Nucleases
CRISPR	Clustered Regularly Interspaced Short Palindrome Repeats
hiPSCs	Human induced Pluripotent Stem Cells
hPSCs	Human Pluripotent stem cells
hESCs	Human Embryonic Stem Cells
HIV-1	Human Immunodeficiency Virus
tracrRNA	trans-activating crRNA
sgRNA	single-guide RNA
IV	Intravenous
saCas9	Staphylococcus aureus Cas9
MPS II	Mucopolysaccharidosis II
IDS	Iduronate 2-Sulfatase
CNS	Central Nervous System
HPV	Human Papillomavirus
HTI	Hereditary Tyrosinemia Type I
AMD	Age-related Macular Degeneration
DMD	Duchenne Muscular Dystrophy
ALS	Amyotrophic Lateral Sclerosis
OTC	Ornithine Transcarbamylase
AAT	Alpha-1 antitrypsin
LCA	Leber’s congenital amaurosis
HR	Homologous Recombination
DTA	Diphtheria A
CCR	Chemokine Co-Receptor
ERT	Enzyme Replacement Therapy
ART	Anti-retroviral therapy
NSG	NOD/SCID/IL2rγnull
SOD1	Superoxide Dismutase 1
rAAV	Recombinant Adeno-Associated Viruses
AAV	Adeno-Associated Viruses
BLT	Bone marrow/liver/thymus
SMA	Spinal Muscular Atrophy
MHC	Major Histocompatibility Complex
PLNP	Polyethylene glycol phospholipid-modified cationic lipid nanoparticle
CNS	Central nervous system
BBB	Blood brain barrier
MOI	Multiplicity of infection
NPs	Nanoparticles
